# Native Flora and Potential Natural Vegetation References for Effective Forest Restoration in Italian Urban Systems

**DOI:** 10.3390/plants14152396

**Published:** 2025-08-02

**Authors:** Carlo Blasi, Giulia Capotorti, Eva Del Vico, Sandro Bonacquisti, Laura Zavattero

**Affiliations:** 1 Interuniversity Research Center “Biodiversity, Ecosystem Services and Sustainability” (CIRBISES), Sapienza University of Rome, 00185 Rome, Italy; carlo.blasi@uniroma1.it (C.B.); sandro.bonacquisti@uniroma1.it (S.B.); laura.zavattero@uniroma1.it (L.Z.); 2 Department of Environmental Biology, Sapienza University of Rome, 00185 Rome, Italy; giulia.capotorti@uniroma1.it

**Keywords:** native species, European Habitats Directive (92/43/EEC), vegetation series, reforestation, urban systems, ecological networks, metropolitan cities

## Abstract

The ongoing decade of UN restoration matches with the European goal of bringing nature back into our lives, including in urban systems, and Nature Restoration Regulation. Within such a framework, this work is aimed at highlighting the ecological rationale and strategic value of an NRRP measure devoted to forest restoration in Italian Metropolitan Cities, and at assessing respective preliminary results. Therefore, the measure’s overarching goal (not to create urban parks or gardens, but activate forest recovery), geographic extent and scope (over 4000 ha and more than 4 million planted trees and shrubs across the country), plantation model (mandatory use of native species consistent with local potential vegetation, density of 1000 seedlings per ha, use of at least four tree and four shrub species in each project, with a minimum proportion of 70% for trees, certified provenance for reproductive material), and compulsory management activities (maintenance and replacement of any dead plants for at least five years), are herein shown and explained under an ecological perspective. Current implementation outcomes were thus assessed in terms of coherence and expected biodiversity benefits, especially with respect to ecological and biogeographic consistency of planted forests, representativity in relation to national and European plant diversity, biogeographic interest and conservation concern of adopted plants, and potential contribution to the EU Habitats Directive. Compliance with international strategic goals and normative rules, along with recognizable advantages of the measure and limitations to be solved, are finally discussed. In conclusion, the forestation model proposed for the Italian Metropolitan Cities proved to be fully applicable in its ecological rationale, with expected benefits in terms of biodiversity support plainly met, and even exceeded, at the current stage of implementation, especially in terms of the contribution to protected habitats. These promising preliminary results allow the model to be recognized at the international level as a good practice that may help achieve protection targets and sustainable development goals within and beyond urban systems.

## 1. Introduction

The European Biodiversity Strategy, along with its Member State level derivations, prompts the return of nature into our lives as a focal and precursory element for achieving the protection of biodiversity, improving the functionality of ecosystems, and enhancing human well-being [1]. Besides vulnerable and/or degraded riparian, forest, and intensive agriculture systems, this overarching objective is also oriented towards urban systems. It is in cities in fact that most people live and are expected to further concentrate in the near future (over 68% by 2050 [2]), and the main contribution to the gross domestic product is generated [3]. On the other hand, however, high levels of air pollution and land consumption/degradation are often reached [4]. Fine particulate matter induced almost 240,000 premature deaths in 2024 in Europe (over 48,000 in Italy alone, according to the European Environment Agency air pollution country fact sheet), while land take for residential, commercial, industrial, and transportation needs is increasingly affecting a considerable amount of species, habitats, and ecosystems at both scales (12% of the habitats and 13% of the species of conservation concern are under severe urbanization pressure in EU [5]; 26% of natural and semi-natural ecosystem types are at risk of collapse due to recent artificialization and fragmentation in Italy [6]), especially within and around cities.

Conservation but also and especially restoration of biodiversity, ecosystems, and their services thus represent a priority for sustainable urban living. Complementary actions are being promoted at the international level with the resolutions of the 15th and 16th Conference of the Parties of the UN Convention on Biological Diversity, concerning a general protection framework for lands, oceans, coastal zones, and inland waters (Global Biodiversity Framework/GBF [7,8], and activities that are consistent with a restoration ecology vision [9,10]. In Europe, a devoted regulation for the restoration of nature has been recently approved (Nature Restoration Regulation/NRR, 2024/1991) that advocates for the recovery of degraded ecosystems, moving from the assessment of their conservation status, and calls for significant elements of nature to be brought back into cities. Restoration measures are therefore expected to improve the quality of both the environment and citizens’ lives, while also contributing to the reduction of greenhouse gases emissions and achievement of climate neutrality by 2050 (EU Climate Law, Regulation 2021/1119 [11]). In Italy, where the protection of biodiversity and ecosystems has been officially included in the Republican Constitution in 2022 (amended Article 9), a special emphasis is being posed on the return of nature into urban systems since the approval of a national Urban Green Strategy in 2018 [12]. This strategy is rooted upon three plain but significant and transversal principles, concerning the need of proper extents to be devoted to nature in cities (“move from square meters to hectares”), proper reference systems to be considered (i.e., “mature forests”), and soil permeability to be improved (even by de-sealing interventions). Concurrently, both Italian and EU forest strategies require native species to be exclusively used in afforestation, reforestation, and tree planting actions [13,14,15,16,17,18].

The National Recovery and Resilience Plan (NRRP), aimed at a socio-economic revitalization after the COVID-19 pandemic especially through ecological and digital transitions, represented an opportunity to operationalize the above-mentioned goals. Namely, within the mission for a “green revolution and ecological transition”, a specific investment was explicitly devoted to the “Protection and enhancement of urban and peri-urban forests”. The target of this investment, already achieved, was to plant at least 4.5 million trees in 4500 hectares across Italian Metropolitan Cities (MCs) [19] for a total amount of 210 million euros of investment.

The first aim of the present work was to highlight the ecological rationale and strategic value of the “Protection and enhancement of urban and peri-urban forests” measure, along with its planning principles, implementation model, and management rules. The second aim was to assess current measure implementation, in terms of ecological coherence and biodiversity benefits. Finally, pros and cons of the measure are discussed, in the light of current strategic goals and normative rules and with respect to science–policy interface reinforcement.

## 2. Material and Methods

### 2.1. Geographic Setting of the Italian NRRP Urban and Peri-Urban Forestation Measure

**Figure 1:**
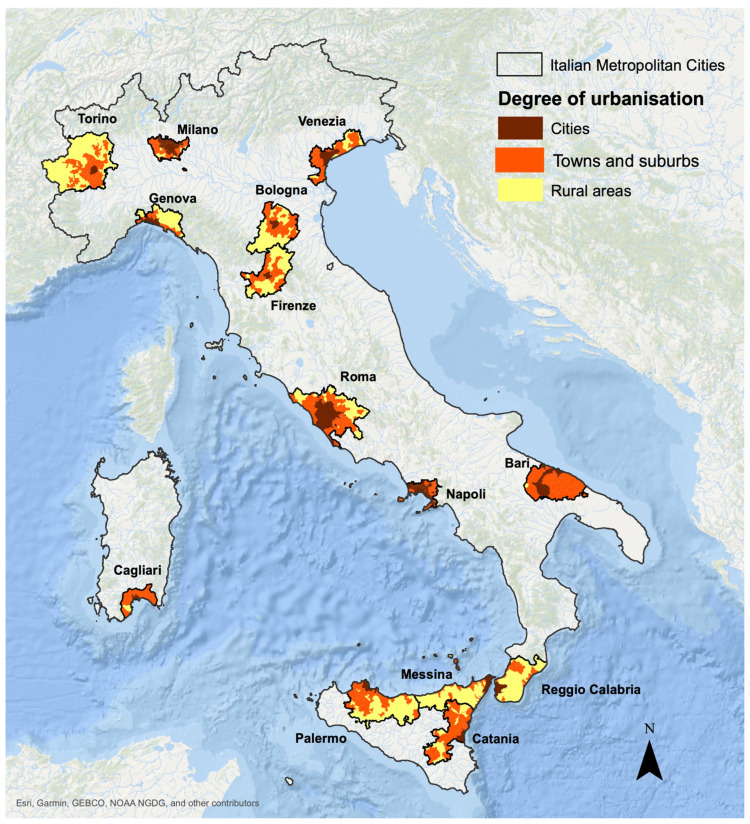
Geographic location of Italian MCs and degree of urbanization (DEGURBA) [20] of included municipalities.

The Italian NRRP measure for the “Protection and enhancement of urban and peri-urban forests” is devoted to the overall national Metropolitan Cities, that are wide administrative entities established in 2014 between the region and the municipal level (Figure 1 and Table 1).

The 14 MCs host more than 21 million inhabitants (36% of the Italian population) and are composed of 1268 individual municipalities, 557 of which (almost 45%) are affected by severe air pollution (i.e., are under infringement proceedings with respect to the EU Directive 2008/50 on ambient air quality and cleaner air). In terms of population size and density (i.e., the degree of urbanization/DEGURBA defined by Eurostat), municipalities within the MCs involve some rural areas and towns and suburbs, besides strict cities with large and dense urban centers. Altogether, they could however be considered as broad urban systems, under a constant and widespread influence of human activities.

Accordingly, the implementation of the measure was based on an original forestation model, with a high diversity of tree and shrub taxa, that is relevant in both urban and extra-urban environments.

### 2.2. Ecological Setting of the Measure

A Forestry Plan, representing the milestone of the NRRP investment, was drawn by a panel of experts including the authors of the present paper, coordinated by the Ministry of the Environment and Energy Security and encompassing public bodies and research institutions (ISTAT/National Institute of Statistic, ISPRA/National Institute for Environmental Protection and Research, CUFAA/Carabinieri Command of Forest, Environmental and Agri-food units, and CIRBISES/Interuniversity Research Centre for “Biodiversity, Ecosystem Services and Sustainability”) [21]. The Plan, conceived as a common technical and scientific reference for the operationalization of the measure, was inspired by the ecological principle of planting “the right tree in the right place and for the right purpose” (i.e., identification and exclusive planting of native woody species that are consistent with local biogeographic and environmental conditions of restoration sites and able to provide needed services) [22]. A preliminary list of suitable phanerophytes and nano-phanerophytes, along with a few geophytes and chamaephytes with a shrubby habit, was therefore provided for each MC. These taxa are consistent with the potential natural vegetation (PNV) types recognized and mapped at the national level [23], which display a forest mature stage and cover more than 5% in the different MCs (Supplementary Material). Whenever appropriate, MCs have been set free to enrich these reference lists according to less representative PNV types and additional local scale knowledge on vegetation series.

Second, the Plan provided preliminary analyses on the state of environment, useful to direct the prioritization of afforestation/reforestation needs with respect to the set of objectives posed by the measure, that are conserving biodiversity and ecosystem services, combating climate change, reducing land consumption and air pollution, and improving the quality of life, well-being, and health of citizens. The analyses included the proportion of sealed soil in each MC, the degree of urbanization of each municipality [20], current tree cover in forests and permanent crops (original CIRBISES processing based on Copernicus HRL data) and current potential for CO_2_ absorption by natural and agricultural systems (Index of foliar area—LAI in winter and summer) (original CIRBISES processing based on Copernicus Global Land Service data).

Finally, the Plan set a common forestation model aimed at guaranteeing biodiversity support, ecological and biogeographic coherence, multifunctionality, resilience, and durable success, by compelling per project:

1. Involvement of an interdisciplinary design group, composed of botanists, foresters, agronomists, ecologists, and natural scientists, along with environmental and landscape planners;
2. Preliminary assessment of the degree of urbanity, degradation, and/or abandonment of putative areas of intervention;
3. Avoidance of tree-line planting and urban gardens projects in favor of right forest stand restoration within urban landscapes, with a minimum extent per area of intervention of at least 3 ha (in a first instance) and 1 ha (in a second instance);
4. Ex-ante floristic and vegetational analysis for the definition of initial conservation status of the areas and local PNV;
5. Selection of at least 4 tree and 4 shrub native species to be adopted in each area;
6. Plantation of 1000 plants per ha under a naturalistic pattern and with a maximum proportion of shrubs of 30% (thus by planting at least 70% of tree species);
7. Certification of provenance of planting material.

Moreover, a set of maintenance and cultivation practices, i.e., replacement of dead seedlings, irrigation, and cleaning of areas, was made mandatory for 5 years after forest plantation, while an ecological monitoring program will be activated after the implementation is completed with respect to biodiversity improvement and protection, CO_2_ absorption, atmospheric PM removal, and soil amelioration. Out of the comprehensive funding, unitary imputable costs for planted hectare/specimens were set uniform for all projects across the involved MCs and mainly devoted to maintenance costs.

### 2.3. Assessment of Ecological Coherence and Expected Biodiversity Benefits of Measure Implementation

The efficacy of the implementation of the above-described forestation model has been assessed in terms of ecological coherence and expected biodiversity benefits, based on the composition and proportion of trees and shrubs effectively adopted by the different MCs (unpublished data provided by the Italian Ministry of the Environment). Especially, foreseen woody plant diversity and consistency with local floras and PNV have been checked and, thus, the lists of planted taxa assessed in terms of representativity, biogeographic, and ecological consistency, and potential contribution to conservation targets.

Nomenclature of taxa provided with the original data was standardized following the checklists of the vascular flora of Italy [24,25]. From these same checklists, adopted plants were also characterized as for taxonomic family of belonging and native/nonnative status, while chorological types were derived from the Flora of Italy [26,27]. Representativity with respect to national and regional woody plant diversity was assessed by comparing the overall list with the native Italian woody flora [28], which also served as a reference for the structural distinction between tree and shrub taxa (scapose and caespitose phanerophytes, respectively), as well as with the Italian dendroflora [29] and the European red list of trees [30]. Consistency of chosen taxa, in both biogeographic (as for distribution) and ecological terms (as for biophysical conditions), was judged with respect to the lists reported in the Forestation Plan, and therefore frequent in forest and shrub stages of the vegetation series [22] in each MC. Biogeographic interest and conservation concern was checked in terms of endemic status [24] and extinction risk, the latter retrieved from the national red list of threatened vascular plants [31] and from the European red lists of trees and endemic shrubs [30,32]. Potential contribution to biodiversity support was finally assessed by comparing the lists with the reference physiognomic combination of habitats of the Directive 92/43/EEC (as described in the Italian Manual of Interpretation of habitats [33]), which represents a reference milestone for the preservation and restoration of nature in Europe.

## 3. Results

The implementation of the Italian NRRP measure for urban and peri-urban forest restoration resulted in the selection of 108 different taxa to be planted across 13 MCs (Table 2). Out of this total number, 52 are trees and 56 shrubs, and all are actually autochthonous, except for the naturalized archaeophyte *Mespilus germanica*. Adopted trees and shrubs include 22% of the native Italian woody flora (composed of 116 trees e 374 shrubs), as well as 41% of the 188 taxa listed in the Italian dendroflora and 18% of the 454 trees listed at the European level.

In taxonomic terms, 11 gymnosperms and 97 angiosperms are included, arranged into 31 families and 62 genera. The most represented families are Rosaceae (with 17 taxa), Fagaceae (12), and Fabaceae (11), while the most represented genera include *Quercus* (8 out of the 11 recognized in Italy), *Pinus* (6), and *Salix* (5). Taxonomic diversity of overall forestation projects reflects that of the entire Italian woody flora, in which Rosaceae (140), Fabaceae (62), Salicaceae (43), Pinaceae, and Fagaceae (18) represent the richest families.

Most adopted trees include five oak species, especially the holm oak (*Quercus ilex*) with nearly 550,000 individuals, and the white oak (*Q. pubescens* subsp. *pubescens*) with over 450,000 individuals (Table 3). Evergreen species are 4 out of the 10 most widely adopted trees, and 7 out of the 10 most widely adopted shrubs.

**Figure 2:**
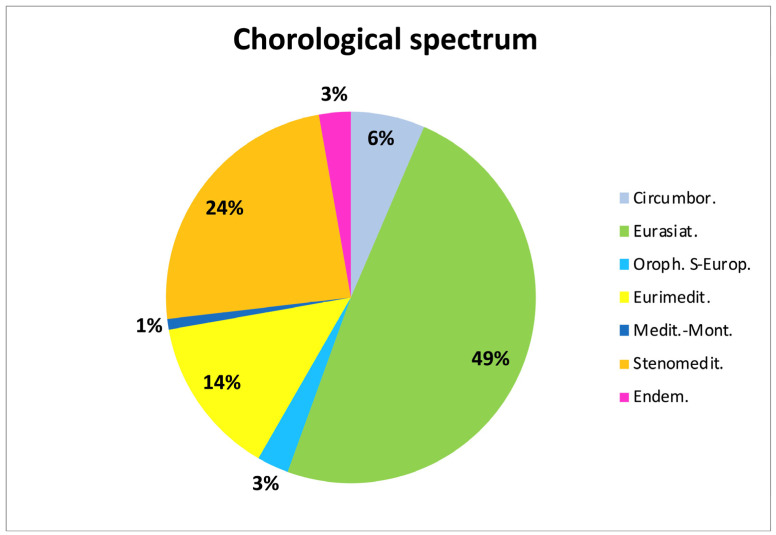
Chorological spectrum of the adopted taxa.

Most of the taxa belong to the Eurasian chorotype (49%), followed by the Mediterranean (39%, 24% of which steno-Mediterranean and 14% euri-Mediterranean), Circumboreal (6%), and South-European orophytes (3%) ones (Figure 2). A single species, *Amelanchier ovalis*, represents the Mediterranean-montane chorotype, while Italian endemytes (3%) include *Alnus cordata*, *Pinus nigra* subsp. *laricio*, and *Genista corsica*. Both the eastern and western components occur, with a prevalence of the former (including for example *Quercus pubescens* subsp. *pubescens* and *Q. frainetto*) that is consistent with the general affinity between the Italian and Balkan floras [34]. Species considered endemic at the European level include instead *Abies alba*, *Alnus cordata*, *Crataegus laevigata*, *Larix decidua*, *Malus sylvestris*, *Pinus cembra*, *Pinus mugo* subsp. *uncinata* (sub *Pinus uncinata*), *Pyrus spinosa*, and, among the shrubs, *Genista corsica*. *Pinus nigra* subsp. *laricio* was not mentioned, probably because Rivers et al. [33] do not refer to the subspecies level.

With respect to the list of trees and shrubs originally suggested with the Forestation Plan, and therefore with respect to the most represented PNV types in each MC, a high consistency emerged for the taxa actually selected for planting (Table 4). In almost all the MCs, over 80% of the suggested taxa have been adopted, ensuring compliance with the biogeographic and ecological guiding principles of the measure. In some cases, namely for Venice and Milan, the taxa suggested in the Plan were exclusively adopted, whilst in other two MCs consistency halted below 60% (i.e., for Palermo and Messina, with 55 and 56%, respectively). In the latter cases, however, alternative choices may be determined according to less represented vegetation series (15 additional vegetation series for Palermo and 8 for Messina) not considered in the Plan due to less than 5% coverage (Table 5).

As an overarching result, the set of trees and shrubs selected with the projects well represented the potential vegetation heterogeneity of the Italian territory in its metropolitan depiction (out of the 279 national potential natural vegetation types—including 240 series and 39 geosigmeta—147 occur in the 13 CM that joined the measure, 125 of which have a forest mature stage and 49 cover more than 5% of the metropolitan land). Along with the above-mentioned cases of Messina and Palermo, in some other MCs this representativity may be also extended towards less widespread ecological potentials.

In terms of risk of extinction *sensu* IUCN, almost all the used taxa are of least concern (LC), except for *Fraxinus excelsior* and *Juniperus turbinata*, judged as near threatened (NT) at the European level, and *Chamaerops humilis* subsp. *humilis*, considered NT in Italy. On the contrary, excluding *Malus sylvestris* and *Paliurus spina-christi*, all the other adopted trees and shrubs could be ascribed to habitats listed under the EU Directive (92/43/EEC), and the main part (66%) belongs to the reference physiognomic composition of priority ones (i.e., in danger of disappearing and for which specific rules are set) (Table 6). Many taxa may occur in several different habitats of the Directive, such as *Fraxinus ornus* subsp. *ornus* (a tree with wide ecological valency, distributed throughout the country, and listed in 11 forest and 2 shrub habitat types), *Acer pseudoplatanus* (11 habitat types), *Fagus sylvatica* (11), *Ostrya carpinifolia* (11), *Sorbus aucuparia* (11), *Pinus sylvestris* (10), *Abies alba* (9), *Fraxinus excelsior* (9), *Quercus petraea* (9), *Acer campestre* (8), and *Quercus robur* (8). Among the shrubs, *Juniperus communis* is the taxon that may occur in the largest number of habitats, spanning from coastal dunes to temperate moorland and shrubs, sub-Mediterranean and temperate shrubs, and forest types. Such a great variety of reference habitats for the juniper is however related to the current taxonomic inclusion under a same species of earlier distinct entities, with different auto-ecology and geographic distribution (i.e., *Juniperus communis* subsp. *alpina*, *J. hemisphaerica*, and *J. nana* are currently considered synonyms). Other shrubs that may occur in manyfold habitats include *Cornus sanguinea*, *Crataegus monogyna*, *Ilex aquifolium*, *Pistacia lentiscus*, *Helichrysum italicum*, and *Rhamnus alaternus* (8 habitat types each). While the belonging of a single taxon to multiple habitats showed floristic and ecological relationships between different settings, the univocal correspondences emerged for 15 taxa revealed instead local biogeographic and environmental peculiarities, as in the case of *Alnus cordata* (exclusively referred to the habitat type “Alluvial forests of *Alnus glutinosa* and *Fraxinus excelsior* of *Alno-Padion*, *Alnion incanae*, *Salicion albae*”), *Ceratonia siliqua* (“*Olea* and *Ceratonia* forests”), *Pinus nigra* subsp. *nigra*, and *P. nigra* subsp. *laricio* (both representative for the priority habitat “(Sub-)Mediterranean pine forests with endemic black pines”), and *Quercus trojana* subsp. *trojana* (“*Quercus trojana* woods”). From the opposite perspective, habitats that comprise in their specific reference combination one or more taxa adopted in MC forestation projects are 76, about 58% of the 132 overall types recognized for Italy, 15 of which are always of priority concern and another one only in the case of gypsy and calcareous substrata [9430(*)]. “*Castanea sativa* woods”, with a nationwide distribution, figure among the habitats related to the largest number of adopted taxa (23), along with the northernmost “Sub-Atlantic and medio-European oak or oak-hornbeam forests of the *Carpinion betuli*” (22 taxa), the central and north-eastern “Illyrian oak-hornbeam forests (*Erythronio-Carpinion*)” (21 taxa), and the nationwide distributed “*Quercus ilex* and *Quercus rotundifolia* forests” (18 taxa).

## 4. Discussion

The focused outline of the Forestation Plan here provided allows the compliance of the Italian NRPP measure “Protection and enhancement of urban and peri-urban forests” with current GBF targets, EU restoration norms, and additional strategic objectives to be checked and highlighted. In particular, notwithstanding unavoidable limitations, the following alignments can be recognized.

First, even though strictly confined to MCs, the overall number of involved municipalities and the nationwide distribution of the measure provide a country-level significance to the network of new planted forests in urban and peri-urban settings. Such features enable the planning and management of overall types of areas, besides those in more natural and rural settings, with the purpose of addressing the driver of land use change (GBF Target 1—Plan and Manage all Areas To Reduce Biodiversity Loss) and help achieve ambitious restoration targets (GBF Target 2—Restore 30% of all Degraded Ecosystems; EU Biodiversity Strategy commitment to plant at least 3 billion additional trees by 2030; EU NRR Article 8- Restoration of urban ecosystems), especially where severe ecosystem degradation occurs due to urbanization dynamics and pollution (GBF Target 7—Reduce pollution to levels that are not harmful to biodiversity) and many people could directly benefit from nature’s contribution (GBF Target 11—Restore, maintain and enhance nature’s contributions to people). Despite this, current limitations in extent and ecoregional representativity would be easily overcome by extending the broadly applicable planning principles, implementation model features, and management rules provided by the Forestation Plan to the other national urban systems.

Second, the requirement for the establishment of true forest ecosystems, rather than tree lined plantations or gardens, concretely addresses the general “bringing nature back into our lives” rationale of the EU Biodiversity Strategy. Especially, notwithstanding a low density of plants is promoted (1000 individuals per hectare) for improving compatibility with other pre-existing natural, archaeological, historical, and landscape values, the minimum intervention areas shall in any case exceed a minimum threshold (at least 1 hectare) to ensure adequate space for the forests that will develop [35] and allow for the establishment of core habitats apart from edges [36]. Moreover, the mandatory and simultaneous adoption of different species of trees and up to 30% of shrubs is in line with the need to support biodiversity besides human well-being (GBF Target 12—Enhance green spaces and urban planning for human well-being and biodiversity) and enables compliance with European guidelines for afforestation and close-to-nature forest management [17,37]. Such compositional, structural, and functional diversity is thus expected to better provide medium- and long-term resilience to restored sites against disturbances and climate change [38], help combine natural and artificial regeneration especially in areas without seed trees [39], and improve the self-regeneration capacity of planted forest stands [40].

Third, the exclusive use of native species that are consistent with PNV allows the measure to effectively counteract the intentional facilitation of alien species (GBF Target 6—Reduce the introduction of invasive alien species by 50% and minimize their impact) [41] and strictly guarantees a combined biogeographic and ecological consistency of new forest stands [42]. Notwithstanding, potential benefits of non-native species are still under debate, especially when they show a marked capacity to provide some ecosystem services [43,44], and different advantages are being recognized to novel ecosystems in cities [45]. Growing scientific evidence is available on the better support for biodiversity and key ecosystem services provision by native forest restoration with respect to other types of tree plantations [46]. Additionally, the mandatory certification for the origin of seeds and seedlings ensures a stricter consistency of adopted plant material at the level of ecotypes, which are particularly important in a country with a marked environmental heterogeneity [47].

Fourth, maintenance and management practices for at least 5 years from planting, compelling replacement of dead seedlings, removal of invasive plants, and subsidy irrigation, have been thought to ensure a high-quality implementation of the measure and its success. Besides these objectives, which are especially critical in Mediterranean contexts due to climate change trends [48], the ongoing care of afforested sites was established by the Italian Forestation Plan as an integral phase of assisted restoration, which falls within the continuum between passive and active interventions [49]. A factual support to the spontaneous recovery of complex and functioning ecosystems after the initial active planting of woody plants is therefore guaranteed, and corrective measures in the case of inappropriate choices of species and/or soil treatment and/or planting patterns can be timely provided. After this phase, the additional monitoring activities devoted to the assessment of ecological benefits will provide instead robust quantitative estimation of cost-effectiveness of the interventions and, eventually, of their observed rather than modeled medium-term outcomes and dynamic trends, in keeping with monitoring requirements of the NRR (Article 20) and as increasingly suggested by restoration ecology theory and ecological restoration practice [50,51].

Fifth, the joint involvement of the national Ministry of the Environment and of local MC administrations, for the respective promotion and implementation of the measure, represents a virtuous example of integrated decision-making about biodiversity (GBF TARGET 14: Integrate biodiversity in decision-making at every level). This interaction allowed in fact to go beyond the conceiving and planning phases of the restoration process towards its operationalization [52], along with a proportionate economic investment (over 200 million euro, in line with the rationale of GBF Target 19: Mobilize $200 billion per year for biodiversity from all sources, including $30 billion through international finance, and with that of the PNRR claiming for adequate investments to be made in restoration), which not only covers 4.5 million plants purchase but especially the maintenance expenses (representing over 90% of the entire investment).

On the other hand, the following observations can emerge from preliminary analyses of implementation results, with respect to plant species and respective number of individuals adopted across the participating MCs. Against a coverage of about 15% of the Italian territory, and considering a prevalent distribution in plain and hilly sectors (almost 75% of the surface of metropolitan municipalities is located below 600 m a.s.l. [53]), the MCs actually selected a representative pool of the national diversity in trees and shrubs at all taxonomic levels. This variety is also valuable at the European scale but, more interestingly, is composed of both deciduous and evergreen species with alternative Eurasiatic or Mediterranean distribution. Overall, such composition guarantees bioclimatic and biogeographic consistency of the new forest network with respect to the marked heterogeneity of the country and provides better chances to adapt and resist to changing climatic conditions at the ecoregional and urban system level [54,55]. Consistency with local biophysical conditions, and therefore with the reference systems provided by PNV types [56], was met as well and in some cases enriched with respect to the species lists suggested by the Plan. These lists may be thus supplemented for future operational enlargement of the forestation model within and beyond the MC contexts, also with the support of nationwide scientific research projects (such as the NRRP National Biodiversity Future Center) and related conceptual and local knowledge updates [57,58].

Besides representativity and consistency, the potential contribution of plantations to biodiversity conservation targets resulted to be more related to the support of habitats than of taxa that are endemic and/or at risk of extinction. As for endemism, which is enlisted among the main criteria for establishing conservation priorities [59], a moderate impact of the forestation measure was originally expected owing to the relatively low proportion of endemics among the woody species in the country (less than 15% [28]). A limited contribution was also found for nationally red-listed species, which however include a limited number of assessed trees and shrubs [31]. By always guaranteeing biogeographic consistency and certified origin of planting material, positive impacts could be again consistently improved through enlarging the measure to include additional urban systems, especially in administrative regions that are intrinsically rich in endemites. Targeted guidance for the forest nursery sector could also give an important input, by making less common and endangered taxa available for restoration projects. Together, these solutions may actively aid halting species extinction and protecting intraspecific genetic diversity (GBF TARGET 4: Halt species extinction, protect genetic diversity, and manage human-wildlife conflicts) but, more interestingly, the consistent number of new forest patches in urban systems could potentially host spontaneous (passive) colonization by additional species of conservation interest in the future. The results already emerged with respect to the habitats of conservation concern, which represent the first target of the EU NRR when not in good condition (Article 4- Restoration of terrestrial, coastal and freshwater ecosystems), could instead be made explicit also in terms of support to threatened ecosystems according to the respective Italian red list [6]. Being national ecosystems typified and assessed on an ecoregional basis [60], the availability of data on precise geographic arrangement of the interventions should however be awaited for such an evaluation. Anyway, the support to nationwide endangered types, such as those joined to alluvial plain and riparian systems [61], can already be stated.

The results demonstrated the expected ecological value of a complex and integrated forestry plan and some of the positive impacts related to its implementation. More in general, the feasibility of a nationwide and ambitious project is being demonstrated, although a number of problems will have to be overcome for next development and enlargement of this forest network. Besides the limitations mentioned above, the recruitment of available sites to accommodate restored urban and peri-urban forests represents a major concern. Planting trees in cities is essential but finding public or private areas to house new forests with lasting permanence is still difficult, as it has been difficult to have all the suggested taxa available and a sufficient number of native plants that are consistent with local PNV types. Both limitations need to be soon solved to enable meeting urban sustainability targets. Additional motivation can be provided by the recognition of these new forests as a network of ‘islands of naturalness’ that will help adapt to climate change and mitigate the effects of urban heat islands, as well as improve the efficiency of existing local ecological networks by means of a new infrastructure dedicated to nature.

To properly advocate these advantages, environmental and socio-economic benefits should thus be measured and not only estimated or modelled. A new phase is therefore opened, supported by the world of research, but also by local authorities and the Ministry of the Environment, aimed at following the growth of planted forests, providing concrete feedback on the choice of species and on the planting model, and activating direct participation of urban dwellers. Definitely, a devoted monitoring plan will help demonstrate that spending to bring back nature in degraded systems is not a cost, but a significant investment to improve both environment and citizen health.

## 5. Conclusions

In conclusion, the forestation model proposed for Italian Metropolitan Cities proved to be fully applicable in its ecological rationale, that is activating actual forest recovery rather than creating urban parks or gardens, with a tangible impact in geographic extent and scope, according to a plantation model able to support native biodiversity in the medium and long term, and under compulsory management activities. At the current level of implementation, the expected benefits have been plainly met in terms of biodiversity support and even exceeded, especially as for the contribution to protected habitats. These promising preliminary results allow the model to be recognized at the international level, as a good practice that may help achieving sustainable development goals and conservation targets within and beyond urban systems, with a special reference to global SDGs and European restoration measures.

## Figures and Tables

**Table 1:** Features of Italian Metropolitan Cities.

Metropolitan City	Population (Number of Inhabitants)	Area (km^2^)	No. of Municipalities Within the Metropolitan City	No. of Municipalities Subject to air Quality Infringement Procedures (2014/2174 and/or 2015/2043; Allegato 1 Decreto Clima 9 October 2020)	No. of Municipalities per DEGURBA/Degree of Urbanization Category (1 = Cities; 2 = Towns and Suburbs; 3 = Rural Areas) [20]		
					1	2	3
Torino	2,208,370	6826.93	312	249	1	114	197
Milano	3,214,630	1575.45	133	134	48	77	8
Venezia	836,916	2472.87	44	44	1	31	12
Genova	817,402	1833.74	67	1	1	26	40
Bologna	1,010,812	3702.27	55	25	1	22	32
Firenze	987,260	3513.64	41	14	1	21	19
Roma	4,216,874	5363.27	121	43	2	55	64
Napoli	2,988,376	1178.92	92	44	50	41	1
Bari	1,226,784	3862.72	41	0	4	36	1
Reggio Calabria	522,127	3210.32	97	0	1	24	72
Palermo	1,208,991	5009.24	82	11	2	32	48
Messina	603,229	3266.08	108	12	1	31	76
Catania	1,077,515	3573.53	58	0	2	42	14
Cagliari	421,688	1248.67	17	0	1	14	2
Total	21,340,974	46,637.65	1268	577	116	566	586

**Table 2:** Adopted plant taxa in Italian Metropolitan Cities projects under the NRRP measure for the “Protection and enhancement of urban and peri-urban forests”.

Clade	Family	Tree (T)/Shrub (S)	Taxon
Gymnosperms	Pinaceae	T	*Abies alba* Mill.
Angiosperms	Sapindaceae	T	*Acer campestre* L.
Angiosperms	Sapindaceae	T	*Acer monspessulanum* L. subsp. *monspessulanum*
Angiosperms	Sapindaceae	T	*Acer opalus* Mill. s.l.
Angiosperms	Sapindaceae	T	*Acer pseudoplatanus* L.
Angiosperms	Betulaceae	T	*Alnus cordata* (Loisel.) Duby
Angiosperms	Betulaceae	T	*Alnus glutinosa* (L.) Gaertn.
Angiosperms	Rosaceae	S	*Amelanchier ovalis* Medik.
Angiosperms	Ericaceae	S	*Arbutus unedo* L.
Angiosperms	Asteraceae	S	*Artemisia arborescens* (Vaill.) L.
Angiosperms	Berberidaceae	S	*Berberis vulgaris* L.
Angiosperms	Betulaceae	T	*Betula pendula* Roth
Angiosperms	Betulaceae	T	*Carpinus betulus* L.
Angiosperms	Betulaceae	T	*Carpinus orientalis* Mill. subsp. *orientalis*
Angiosperms	Fagaceae	T	*Castanea sativa* Mill.
Angiosperms	Cannabaceae	T	*Celtis australis* L. subsp. *australis*
Angiosperms	Fabaceae	T	*Ceratonia siliqua* L.
Angiosperms	Fabaceae	T	*Cercis siliquastrum* L. subsp. *siliquastrum*
Angiosperms	Arecaceae	T	*Chamaerops humilis* L. subsp. *humilis*
Angiosperms	Cistaceae	S	*Cistus monspeliensis* L.
Angiosperms	Cistaceae	S	*Cistus salviifolius* L.
Angiosperms	Fabaceae	S	*Colutea arborescens* L.
Angiosperms	Cornaceae	T	*Cornus mas* L.
Angiosperms	Cornaceae	S	*Cornus sanguinea* L.
Angiosperms	Betulaceae	T	*Corylus avellana* L.
Angiosperms	Rosaceae	S	*Crataegus laevigata* (Poir.) DC.
Angiosperms	Rosaceae	S	*Crataegus monogyna* Jacq.
Angiosperms	Fabaceae	S	*Cytisus scoparius* (L.) Link
Angiosperms	Fabaceae	S	*Cytisus villosus* Pourr.
Angiosperms	Fabaceae	S	*Emerus major* Mill.
Angiosperms	Ericaceae	S	*Erica arborea* L.
Angiosperms	Ericaceae	S	*Erica scoparia* L.
Angiosperms	Celastraceae	S	*Euonymus europaeus* L.
Angiosperms	Euphorbiaceae	S	*Euphorbia dendroides* L.
Angiosperms	Fagaceae	T	*Fagus sylvatica* L.
Angiosperms	Rhamnaceae	S	*Frangula alnus* Mill.
Angiosperms	Oleaceae	T	*Fraxinus angustifolia* Vahl subsp. *oxycarpa* (M.Bieb. ex Willd.) Franco & Rocha Afonso
Angiosperms	Oleaceae	T	*Fraxinus excelsior* L.
Angiosperms	Oleaceae	T	*Fraxinus ornus* L. subsp. *ornus*
Angiosperms	Fabaceae	S	*Genista corsica* (Loisel.) DC.
Angiosperms	Fabaceae	S	*Genista monspessulana* (L.) L.A.S.Johnson
Angiosperms	Asteraceae	-	*Helichrysum italicum* (Roth) G.Don
Angiosperms	Aquifoliaceae	S	*Ilex aquifolium* L.
Gymnosperms	Cupressaceae	S	*Juniperus communis* L.
Gymnosperms	Cupressaceae	S	*Juniperus oxycedrus* L.
Gymnosperms	Cupressaceae	S	*Juniperus turbinata* Guss.
Gymnosperms	Pinaceae	T	*Larix decidua* Mill.
Angiosperms	Lauraceae	S	*Laurus nobilis* L.
Angiosperms	Oleaceae	S	*Ligustrum vulgare* L.
Angiosperms	Rosaceae	T	*Malus sylvestris* (L.) Mill.
Angiosperms	Rosaceae	T	*Mespilus germanica* L.
Angiosperms	Myrtaceae	S	*Myrtus communis* L.
Angiosperms	Apocynaceae	S	*Nerium oleander* L. subsp. *oleander*
Angiosperms	Oleaceae	T	*Olea europaea* L. var. *sylvestris*
Angiosperms	Betulaceae	T	*Ostrya carpinifolia* Scop.
Angiosperms	Rhamnaceae	S	*Paliurus spina-christi* Mill.
Angiosperms	Oleaceae	S	*Phillyrea angustifolia* L.
Angiosperms	Oleaceae	S	*Phillyrea latifolia* L.
Gymnosperms	Pinaceae	T	*Pinus cembra* L.
Gymnosperms	Pinaceae	T	*Pinus halepensis* Mill. subsp. *halepensis*
Gymnosperms	Pinaceae	T	*Pinus nigra* J.F.Arnold subsp. *nigra*
Gymnosperms	Pinaceae	T	*Pinus nigra* J.F.Arnold subsp. *laricio* Palib. ex Maire
Gymnosperms	Pinaceae	T	*Pinus sylvestris* L.
Gymnosperms	Pinaceae	S	*Pinus mugo* Turra subsp. *uncinata* (Ramond ex DC.) Domin
Angiosperms	Anacardiaceae	S	*Pistacia lentiscus* L.
Angiosperms	Anacardiaceae	S	*Pistacia terebinthus* L. subsp. *terebinthus*
Angiosperms	Salicaceae	T	*Populus alba* L.
Angiosperms	Salicaceae	T	*Populus nigra* L. subsp. *nigra*
Angiosperms	Salicaceae	T	*Populus tremula* L.
Angiosperms	Rosaceae	T	*Prunus avium* (L.) L.
Angiosperms	Rosaceae	S	*Prunus mahaleb* L.
Angiosperms	Rosaceae	S	*Prunus padus* L.
Angiosperms	Rosaceae	S	*Prunus spinosa* L. subsp. *spinosa*
Angiosperms	Rosaceae	T	*Pyrus spinosa* Forssk.
Angiosperms	Rosaceae	T	*Pyrus communis* L. subsp. *pyraster* (L.) Ehrh.
Angiosperms	Fagaceae	T	*Quercus cerris* L.
Angiosperms	Fagaceae	T	*Quercus frainetto* Ten.
Angiosperms	Fagaceae	T	*Quercus ilex* L.
Angiosperms	Fagaceae	T	*Quercus petraea* (Matt.) Liebl.
Angiosperms	Fagaceae	T	*Quercus pubescens* Willd. subsp. *pubescens*
Angiosperms	Fagaceae	T	*Quercus robur* L.
Angiosperms	Fagaceae	T	*Quercus suber* L.
Angiosperms	Fagaceae	T	*Quercus trojana* Webb subsp. *trojana*
Angiosperms	Rhamnaceae	S	*Rhamnus alaternus* L. subsp. *alaternus*
Angiosperms	Rhamnaceae	S	*Rhamnus cathartica* L.
Angiosperms	Rosaceae	S	*Rosa canina* L.
Angiosperms	Rosaceae	S	*Rosa sempervirens* L.
Angiosperms	Lamiaceae	S	*Salvia rosmarinus* Spenn.
Angiosperms	Asparagaceae	-	*Ruscus aculeatus* L.
Angiosperms	Salicaceae	T	*Salix alba* L.
Angiosperms	Salicaceae	T	*Salix caprea* L.
Angiosperms	Salicaceae	S	*Salix eleagnos* Scop.
Angiosperms	Salicaceae	S	*Salix purpurea* L. subsp. *purpurea*
Angiosperms	Salicaceae	S	*Salix triandra* L. subsp. *triandra*
Angiosperms	Viburnaceae	S	*Sambucus nigra* L.
Angiosperms	Rosaceae	S	*Sorbus aria* (L.) Crantz
Angiosperms	Rosaceae	T	*Sorbus aucuparia* L.
Angiosperms	Rosaceae	T	*Sorbus domestica* L.
Angiosperms	Rosaceae	S	*Sorbus torminalis* (L.) Crantz
Angiosperms	Fabaceae	S	*Spartium junceum* L.
Angiosperms	Tamaricaceae	T	*Tamarix africana* Poir.
Angiosperms	Lamiaceae	S	*Teucrium fruticans* L. subsp. *fruticans*
Angiosperms	Malvaceae	T	*Tilia cordata* Mill.
Angiosperms	Lamiaceae	-	*Thymbra capitata* (L.) Cav.
Angiosperms	Ulmaceae	T	*Ulmus minor* Mill.
Angiosperms	Viburnaceae	S	*Viburnum lantana* L.
Angiosperms	Viburnaceae	S	*Viburnum opulus* L.
Angiosperms	Viburnaceae	S	*Viburnum tinus* L. subsp. *tinus*

**Table 3:** Plant taxa adopted in afforestation projects with the largest numbers of individuals across overall MCs. Leaf-shedding habits of the plants (E = Evergreen; D = Deciduous; (1) = Deciduous but with year-round photosynthesizing stems) are also reported.

	Leaf-Shedding Habits	N° of Individuals	N° of Adopting MCs
**Tree taxa**			
*Quercus ilex* L.	E	548,934	10
*Quercus pubescens* Willd. subsp. *pubescens*	D	467,211	11
*Fraxinus ornus* L. subsp. *ornus*	D	337,694	10
*Quercus cerris* L.	D	230,224	7
*Quercus suber* L.	E	225,896	7
*Acer campestre* L.	D	129,839	10
*Olea europaea* L. var. *sylvestris*	E	115,051	5
*Quercus robur* L.	D	110,749	6
*Populus alba* L.	D	103,933	8
*Ceratonia siliqua* L.	E	102,949	6
**Shrub taxa**			
*Crataegus monogyna* Jacq.	D	122,885	12
*Spartium junceum* L.	(1)	107,097	7
*Prunus spinosa* L. subsp. *spinosa*	D	89,844	9
*Pistacia lentiscus* L.	E	88,293	9
*Myrtus communis* L.	E	66,253	9
*Arbutus unedo* L.	E	62,473	10
*Ligustrum vulgare* L.	D	49,256	5
*Erica arborea* L.	E	41,817	7
*Phillyrea latifolia* L.	E	35,991	9
*Viburnum tinus* L. subsp. *tinus*	E	35,181	8

**Table 4:** Number of plant taxa suggested in the Forestation Plan, number of plant taxa selected for planting in each MC, and their proportional consistency with the Forestation Plan.

	No. of Forestation Plan Suggested Taxa for MC	No. of Adopted Taxa	No. of Adopted Taxa Consistent with the Forestation Plan	% of Adopted Taxa Consistent with the Forestation Plan
Bari	34	26	21	81
Cagliari	36	29	26	90
Catania	50	24	20	83
Firenze	56	33	29	88
Genova	53	32	27	84
Messina	34	50	28	56
Milano	39	23	23	100
Napoli	55	54	45	83
Palermo	42	40	22	55
Reggio Calabria	39	31	28	90
Roma	65	54	50	93
Torino	53	46	38	83
Venezia	38	17	17	100

**Table 5:** Vegetation series types intercepted by the Italian MCs. Besides the overall number of occurring types (first column), which include also the series and geosigmeta with shrub or herbaceous mature stages, the number of those with a forest mature stage, which represent reference systems for the forestation projects, are reported (second column). Among the latter, the number of more widespread types (with at least 5% cover) and their respective share in each MC, which informed the preliminary list of suitable taxa provided by the Forestation Plan, are shown (third and fourth column, respectively).

	No. of Total Vegetation Series/ Geosigmeta Occurring in the MC	No. of Vegetation Series/ Geosigmeta with Forest Mature Stage	No. of Vegetation Series/Geosigmeta with a Forest Mature Stage and at Least 5% Coverage	Proportion of MC Area (%) Interested by Vegetation Series/Geosigmeta with a Forest Mature Stage and at Least 5% Coverage
Bari	10	9	4	99.3
Cagliari	12	10	4	79.9
Catania	18	14	5	81.8
Firenze	20	19	7	87.9
Genova	16	16	6	92.8
Messina	12	10	4	81
Milano	6	6	3	90.6
Napoli	13	10	4	88.8
Palermo	23	20	7	82.4
Reggio Calabria	17	13	4	78.9
Roma	25	22	7	82.8
Torino	23	18	5	67.1
Venezia	5	2	2	70.6
Total	147	125	49	-

**Table 6:** Adopted taxa and habitats listed under the EU Directive (92/43/EEC) to which they could be ascribed (see the relative reference physiognomic combination of the Italian Manual of Interpretation [33]). The asterisks indicate priority natural habitat types under the Directive, which are in danger of disappearance and for the conservation of which the European Community has a particular responsibility. The asterisks between brackets indicate that the priority importance is conditioned by peculiar habitat characteristics.

Taxon	Habitat in Which the Taxon is Indicated in the Reference Physiognomic Combination																
	1	2	3	4	5	6	7	8	9	10	11	12	13	14	15	16	17
*Abies alba* Mill.	9110	9130	9140	91K0	9210	9220 *	9410	9420	9510 *								
*Acer campestre* L.	5230 *	9150	9160	9170	9180 *	91E0 *	91L0	9260									
*Acer monspessulanum* L. subsp. *monspessulanum*	9250	9580 *															
*Acer opalus* Mill. s.l. ^1^	9150	9180 *	91K0	91L0	9260												
*Acer pseudoplatanus* L.	9130	9140	9160	9180 *	91E0 *	91K0	91L0	9210	9220 *	9260	9410						
*Alnus cordata* (Loisel.) Duby	91E0 *																
*Alnus glutinosa* (L.) Gaertn.	9180 *	91B0	91E0 *	91F0													
*Amelanchier ovalis* Medik.	4070 *	5110	9150	91H0 *	9530 *												
*Arbutus unedo* L.	2270 *	9330	9340														
*Artemisia arborescens* (Vaill.) L.	1430	5220 *															
*Berberis vulgaris* L.	5110	5130	9150	91H0 *	9410												
*Betula pendula* Roth	4030	9110	9190	9260													
*Carpinus betulus* L.	5230 *	9160	9170	9180 *	91AA*	91L0	9260										
*Carpinus orientalis* Mill. subsp. *orientalis*	91AA *	91M0	9250														
*Castanea sativa* Mill.	9110	9120	9190	91L0	9260												
*Celtis australis* L. subsp. *australis*	5230 *	9340															
*Ceratonia siliqua* L.	9320																
*Cercis siliquastrum* L. subsp. *siliquastrum*	9340																
*Chamaerops humilis* L. subsp. *humilis*	2250 *	2260	5210	5220 *	5330	5420	9320										
*Cistus monspeliensis* L.	2260	5420	9330														
*Cistus salviifolius* L.	4030	5330	9330														
*Colutea arborescens* L.	5330	91H0 *															
*Cornus mas* L.	5110	91H0 *	91L0														
*Cornus sanguinea* L.	5130(1)	9150	9160	9170	91AA *	91B0	91F0	9340									
*Emerus major* Mill.	5110	5330	9150	9160	91AA *												
*Corylus avellana* L.	9160	9170	9180 *	91K0	91L0	9260	9410										
*Crataegus laevigata* (Poir.) DC.	9170	91L0															
*Crataegus monogyna* Jacq.	5110	5130	9160	9170	91AA *	91B0	91L0	9340									
*Cytisus scoparius* (L.) Link	4030	9170	9260														
*Cytisus villosus* Pourr.	9330																
*Erica arborea* L.	2260	4030	5330	9330	9340												
*Erica scoparia* L.	4030	9330															
*Euonymus europaeus* L.	9160	91M0	92A0														
*Euphorbia dendroides* L.	5210	5220 *	5330	9320													
*Fagus sylvatica* L.	9110	9120	9130	9140	9150	9170	91K0	9210	9220 *	9260	9410						
*Frangula alnus* Mill.	4030	4070 *	9160	9190	91D0 *	91E0 *	9260										
*Fraxinus angustifolia* Vahl subsp. *oxycarpa* (M.Bieb. ex Willd.) Franco & Rocha Afonso ^2^	9160	91B0	91E0 *	91F0	91L0	92A0	92C0										
*Fraxinus excelsior* L.	9130	9160	9170	9180 *	91E0 *	91F0	91L0	9260	9410								
*Fraxinus ornus* L. subsp. *ornus*	4070 *	5230 *	9150	9160	9170	9180 *	91AA *	91H0 *	91L0	91M0	9250	9260	9340				
*Genista corsica* (Loisel.) DC.	5420	5430															
*Genista monspessulana* (L.) L.A.S. Johnson	9330																
*Helichrysum italicum* (Roth) G.Don ^3^	2210	2260	3250	4090	5320	5330	5420	5410									
*Ilex aquifolium* L.	9110	9120	91E0 *	91L0	9210	9220 *	9380	9580 *									
*Juniperus communis* L. ^4^	2160	2250 *	4030	4060	4070 *	4070 *	4080	5110	5130	9220 *	9410	9420	9430 *	9510 *	9530 *	9560 *	95A0
*Juniperus oxycedrus* L. ^5^	2250 *	2270 *	5110	5210	5330	9540	9560 *										
*Juniperus turbinata* Guss. ^6^	2250 *	2270 *	5330														
*Larix decidua* Mill.	9110	9140	91K0	9410	9420	9430 *											
*Laurus nobilis* L.	5230 *	91B0	92A0	9340													
*Ligustrum vulgare* L.	5110	9150	9170	91AA *	91L0	91M0	9340										
*Malus sylvestris* (L.) Mill.	_																
*Mespilus germanica* L.	91M0																
*Myrtus communis* L.	2250 *	5210	5330	9320	9330												
*Nerium oleander* L. subsp. *oleander*	5420	92C0	92D0														
*Olea europaea* L. var. *sylvestris*	2260	5210	5220 *	5330	9320												
*Ostrya carpinifolia* Scop.	5230 *	9110	9150	9180 *	91AA *	91L0	9260	9340	9380	4070 *	91K0						
*Paliurus spina-christi* Mill.	_																
*Phillyrea angustifolia* L.	2250 *	2260	2270 *	5220 *	9320	9330	9340										
*Phillyrea latifolia* L.	5210	5220 *	5310	9340	2250 *	2260											
*Pinus cembra* L.	9410	9420	9430 *														
*Pinus halepensis* Mill. subsp. *halepensis*	2270 *	9540															
*Pinus* nigra J.F.Arnold subsp. *nigra*	9530 *																
*Pinus nigra* J.F.Arnold subsp. *laricio* Palib. ex Maire	9530 *																
*Pinus sylvestris* L.	3240	4030	9110	9150	9160	9190	91D0 *	91H0 *	9410	9430 *							
*Pinus mugo* Turra subsp. *uncinata * (Ramond ex DC.) Domin ^7^	91D0 *	9410	9420	9430 *													
*Pistacia lentiscus* L.	2250 *	2260	2270 *	5210	5220 *	5330	5420	9320									
*Pistacia terebinthus* L. subsp. *terebinthus*	5110	5220 *	9340														
*Populus alba* L.	3280	5230 *	92A0														
*Populus nigra* L. subsp. *nigra*	3230	3280	91E0 *	91F0	92A0												
*Populus tremula* L.	4030	9180 *	9190	91F0	9260	92A0	9410										
*Prunus avium* (L.) L.	9130	9160	9170	9180 *	91L0	9260											
*Prunus mahaleb* L.	5110	91H0 *															
*Prunus padus* L.	9160	91F0															
*Prunus spinosa* L. subsp. *spinosa*	5110	5130															
*Pyrus spinosa* Forssk.	9330																
*Pyrus communis* L. subsp. *pyraster* (L.) Ehrh.	91H0 *																
*Quercus cerris* L.	9160	91H0 *	91K0	91L0	91M0	9260	9340										
*Quercus frainetto* Ten.	91M0	9330															
*Quercus ilex* L.	5110	5230 *	5310	6310	9330	9340											
*Quercus petraea* (Matt.) Liebl.	4030	9110	9120	9160	9170	9190	91L0	91M0	9260								
*Quercus pubescens* Willd. subsp. *pubescens*	9150	91AA *	91H0 *	9260													
*Quercus robur* L.	9110	9120	9160	9170	9180 *	9190	91F0	91L0									
*Quercus suber* L.	6310	9330	9340														
*Quercus trojana* Webb subsp. *trojana*	9250																
*Rhamnus alaternus* L. subsp. *alaternus*	2250 *	2260	2270 *	5210	5220 *	5330	9320	9340									
*Rhamnus cathartica* L.	2160																
*Rosa canina* L.	5130																
*Rosa sempervirens* L.	91AA *	9250	92A0														
*Salvia rosmarinus* Spenn.	2260	5330	5420														
*Ruscus aculeatus* L.	2250 *	5230 *	5310	91H0 *	9260	9120	9160										
*Salix alba* L. ^8^	3280	91E0 *	92A0	92C0													
*Salix caprea* L.	4030																
*Salix eleagnos* Scop. ^8^	3220	3230	3240	3280													
*Salix purpurea* L. subsp. *purpurea* ^8^	3220	3230	3240	3280													
*Salix triandra* L. subsp. *triandra* ^8^	3240	3280															
*Sambucus nigra* L.	9160	91E0 *	91F0	9260	92A0												
*Sorbus aria* (L.) Crantz	4070 *	9110	9150	9260	9410	9580 *											
*Sorbus aucuparia* L. ^9^	4070 *	9110	9120	9130	9140	9190	91D0 *	91K0	9220 *	9410	9420						
*Sorbus domestica* L.	9170	91H0 *															
*Sorbus torminalis* (L.) Crantz	9150	91H0 *	91L0	9260	9170												
*Spartium junceum* L.	5420	92D0															
*Tamarix africana* Poir.	92D0																
*Teucrium fruticans* L. subsp. *fruticans*	5220 *	5330	5420														
*Tilia cordata* Mill.	9110	9160	9170	9180 *	9260												
*Thymbra capitata* (L.) Cav.	2260	5330	5420														
*Ulmus minor* Mill.	5230 *	5310	91B0	91E0 *	91F0	91L0											
*Viburnum lantana* L.	4070 *	5110	9150														
*Viburnum opulus* L.	9160	91E0 *	91F0														
*Viburnum tinus* L. subsp. *tinus*	5310	5330	9340														

^1^ By considering also *Acer opalus* subsp. *opalus*, *A. opalus* subsp. *obtusatum*, *A. obtusatum*, *A. opulifolium*, *A. obtusatum* subsp. *neapolitanum*. ^2^ Sub *Fraxinus angustifolia* in 91E0 * e 91F0. ^3^ By considering also *Helichrysum italicum* subsp. *microphyllum*, *H. italicum* subsp. *italicum*, *H. microphyllum*. ^4^ By considering also *Juniperus communis* subsp. *alpina*, *J. hemisphaerica*, *J. nana*, *J. alpina* subsp. *nana*. ^5^ By considering also *Juniperus oxycedrus* subsp. *macrocarpa* in 2250 * and 2270 *. ^6^ By considering also *Juniperus phoenicea* subsp. *turbinata*, *J. phoenicea*. ^7^ Habitats with reference to *Pinus mugo* are excluded. ^8^ Cited as *Salix* sp. pl. in 3280. ^9^ By considering also *Sorbus aucuparia* subsp. *praemorsa* in 9220 *.

## Data Availability

The original contributions presented in this study are included in the article. Further inquiries can be directed to the corresponding author.

## Supplementary Materials

The following supporting information can be downloaded at: https://www.mdpi.com/article/10.3390/plants14152396/s1, Table S1: Vegetation Series/Geosigmeta considered representative in each MC. Names and related codes referto Blasi 2010 (ed.).
